# Single-Cell RNA Sequencing of Cerebrospinal Fluid as an Advanced Form of Liquid Biopsy for Neurological Disorders

**DOI:** 10.3390/brainsci12070812

**Published:** 2022-06-22

**Authors:** Anudeep Yekula, Jovanna Tracz, Jordina Rincon-Torroella, Tej Azad, Chetan Bettegowda

**Affiliations:** 1 Department of Surgery, Yale School of Medicine, New Haven, CT 06510, USA; anudeep.yekula@yale.edu; 2 Department of Neurosurgery, Johns Hopkins University School of Medicine, Baltimore, MD 21287, USA; jtracz1@jhmi.edu (J.T.); jrincon2@jhmi.edu (J.R.-T.); tazad1@jhmi.edu (T.A.)

**Keywords:** scRNA sequencing, single-cell RNA sequencing, RNA-Seq, transcriptome, neurological disease, neuroinflammation

## Abstract

Diagnosis and longitudinal monitoring of neurological diseases are limited by the poor specificity and limited resolution of currently available techniques. Analysis of circulating cells in cerebrospinal fluid (CSF) has emerged as a promising strategy for the diagnosis, molecular characterization, and monitoring of neurological disease. In comparison to bulk sequencing analysis, single-cell sequencing studies can provide novel insights into rare cell populations and uncover heterogeneity in gene expression at a single-cell resolution, which has several implications for understanding disease pathology and treatment. Parallel development of standardized biofluid collection protocols, pre-processing strategies, reliable single-cell isolation strategies, downstream genomic analysis, and robust computational analysis is paramount for comprehensive single-cell sequencing analysis. Here we perform a comprehensive review of studies focusing on single-cell sequencing of cells in the CSF of patients with oncological or non-oncological diseases of the central nervous system.

## 1. Introduction

The current approach to diagnosing and monitoring neurological diseases is largely reliant on imaging and tissue biopsy. Imaging does not provide molecular or biological information regarding diagnosis, response to therapy, or progression of the disease. Furthermore, obtaining tissue samples in the central nervous system (CNS) is not always feasible owing to the location of the lesion, disease type, and potential complications. Longitudinal tissue biopsies in the context of the brain are very challenging, again due to the inherent risks of neurosurgical biopsy [1].

A liquid biopsy involves the study of analytes (e.g., circulating cells, cell-free DNA or extracellular vesicles) in biofluids (typically blood or cerebrospinal fluid [CSF]) to allow for the diagnosis and monitoring of disease. Analysis of circulating cells has evolved as a promising strategy to study neurological diseases. Combining circulating cell analysis with advanced sequencing methodologies such as single-cell RNA sequencing (scRNA-seq) can provide valuable insights into disease biology and immunology, diagnosis, and response to therapy. CSF, already considered a biofluid of high significance in the diagnosis of CNS pathologies due to its proximity, accessibility, and role in immunosurveillance, holds unique cell populations which can be analyzed to improve our understanding of the pathology, diagnosis, and treatment of CNS disease. In this review, we discuss emerging studies exploring scRNA-seq of circulating cells in CSF to investigate oncological and non-oncological diseases of the CNS.

## 2. CSF as a Repertoire of Biomarkers in Diseases of the CNS

CSF surrounds the brain and the spinal cord and plays a vital role in CNS homeostasis [2]. Τhere are typically very few cells in the CSF; usually on the order of 1–5 cells/μL [3]. CSF is comprised of select immune cells such as blood-derived lymphocytes and monocytes in addition to macromolecules such as antibodies that cross the blood-CSF barrier at the choroid plexus. The function of such immune cells, which are predominantly central memory CD4+ T cells, is immunosurveillance [4,5,6]. The glymphatic system—the lymphatic system of the CNS—carries immune cells within CSF from brain meningeal lymphatic vessels to deep cervical lymph nodes, enabling peripheral T-cells to encounter and respond to antigens present within the brain [6,7].

CSF is considered a biofluid of high significance in the diagnosis of CNS pathologies due to its proximity, accessibility, and role in immunosurveillance. In several neurological diseases, the CSF composition, opacity, and opening pressure can be altered. By measuring various CSF components using methodologies such as cell counts, cytology, staining, culture, immunoglobulins, and lactate levels, the diagnosis, severity and prognostication of conditions including infection, subarachnoid hemorrhage (SAH), and autoimmune demyelinating conditions can be made [8]. Additionally, obtaining CSF via lumbar puncture is relatively easy to perform, even in resource-limited settings [9].

CSF pleocytosis is a hallmark of neuroinflammation commonly seen in meningitis, autoimmune diseases, and leptomeningeal disease [10]. For instance, the detection of neoplastic cells within CSF indicates the leptomeningeal spread of cancer. CSF pleocytosis may also result from non-inflammatory etiologies such as neurosurgical intervention, acute SAH, or blood contamination during a lumbar puncture. For instance, elevated erythrocyte/siderophage numbers on cytoslides are common in patients with SAH [7]. Reactive CSF pleocytosis is characterized by mild to moderately increased cell count with non-activated mononuclear cells and a small fraction of neutrophils. Massive granulocytosis (>1000 cells/μL) is seen in bacterial meningitis, and mononuclear pleocytosis with activated lymphocytes and monocytes is seen in viral meningitis. Aseptic bacterial meningitis secondary to less common pathogens such as *Mycobacterium tuberculosis*, *Listeria monocytogenes*, *Treponema Pallidum*, or *Borrelia burgdorferi* also results in mononuclear pleocytosis [10]. Autoimmune diseases of the CNS such as Multiple Sclerosis and Autoimmune Encephalitis have lymphocyte-predominant pleocytosis [10].

Recent studies in the neuro-oncology space have pioneered and demonstrated the feasibility of studying various liquid biopsy analytes in primary and metastatic brain tumors [11,12,13,14,15,16,17,18,19]. Studying circulating cells in CSF has always been an area of significant interest for the diagnosis and monitoring of several neurological diseases. Combining this methodology with advanced sequencing modalities can provide novel insights with the potential to revolutionize the care of CNS diseases.

## 3. Single-Cell Sequencing of CSF

While traditional bulk transcriptomic sequencing provides an average expression signal for an ensemble of cells, there is increasing evidence in tissue analysis that heterogeneity in gene expression exists even among very similar cell types, driving different cell fates [20]. Pioneering single-cell studies by James Eberwine et al. [21] used linear amplification of individual cell complementary DNAs for in vitro transcription and PCR-based exponential amplification, laying the foundation for the era of scRNA-seq. This technology allowed us to identify genetic signatures of both neoplastic cells and rare outlier cell populations, which has several implications for targeted treatments [22].

**Figure 1:**
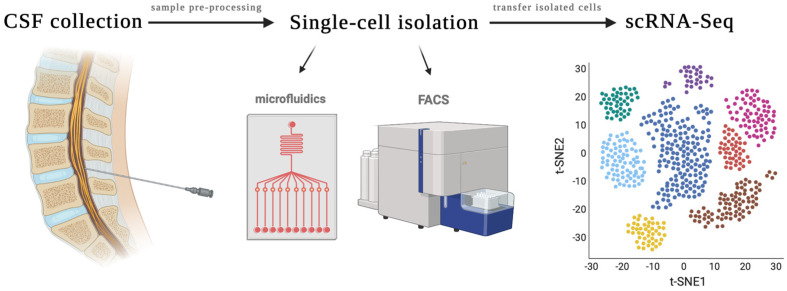
Overview of single-cell RNA Sequencing of the CSF. CSF is obtained via lumbar puncture. The sample is then pre-processed for single cell isolation. Methods for single-cell isolation include microfluidics, fluorescence-activated cell sorting (FACS), flow cytometry, pico-wells, and laser microdissection. Isolated cells are then transferred for scRNA-Seq, which analyzes transcriptomes on a cell-by-cell basis and, depending on sequencing technology used, may prepare barcoded, next-generation sequencing complementary DNA libraries. One method by which data can be visualized is with a t-distributed stochastic neighbor embedding (t-SNE) plot, which may apply a clustering algorithm to visualize cells with similar genetic signatures.

Researchers have applied such advances in single-cell analysis to the study of circulating cells in the CSF (Figure 1). Parallel development of standardized biofluid collection protocols, pre-processing strategies, reliable single-cell isolation strategies, downstream genomic analysis technologies, and robust computational analysis is paramount to the success of liquid biopsy using circulating cells in CSF for the diagnosis and treatment of neurological diseases. Understanding the nuances of the single-cell liquid biopsy pipeline is critical in developing clinically translatable assays that can impact patient care from a diagnostic, prognostic, or therapeutic standpoint.

### 3.1. Sample Pre-Processing

For all liquid biopsy-based assays, including single-cell assays, reliable sample collection and preprocessing is necessary. The volume of CSF required for adequate single-cell isolation may vary depending on disease-specific quantities of cells present in the CSF. For instance, circulating tumor cells (CTCs) are extremely rare and thus may require larger volumes of CSF to be collected for adequate analysis in comparison to the smaller volumes needed to study infectious diseases [23]. Immediate sample processing is preferred for circulating cell analysis to preserve cell viability which can impact the transcriptomic landscape of cells [1]. Robust study design, appropriate sample sizes with substantial statistical power, and proper positive and negative controls are all crucial in downstream analysis. Additionally, secondary factors such as patient age, sex, comorbidities, medications, co-existing neurological conditions, location and the extent of disease, extent of BBB damage, timing and method of the sample collection, quality of sample collected, and sample handling and storage conditions pre-analysis can all potentially impact the discovery of biomarkers [1]. Standardized upstream protocols are essential for high-quality downstream analysis. Well-documented, paired clinical data are important to allow for clinical correlations [24].

Although CSF is an ideal biofluid from an anatomic proximity standpoint, emerging studies have shown that other easily obtainable biofluids such as blood/plasma also have circulating cells of interest. However, blood contains an abundance of normal cells, and isolating disease-specific circulating cells in blood has several inherent limitations [23]. Parallel comparison of blood and CSF to study circulating cells can objectively demonstrate the ideal biofluid for maximal cell recovery. Standardized collection protocols, well-balanced patient and control cohorts that include several clinical sites with patient demographics, pre-analytic data variables and a good gender and racial balance provide the study with an increased likelihood of obtaining a clinically significant cellular signature [25].

### 3.2. Single-Cell Isolation Techniques

Isolating single cells from CSF is the next step in the single-cell transcriptomic analysis pathway. Arguably, this is the greatest source of technical variation, contamination, and batch effect in any single cell study [26]. Unlike single-cell isolation from tissues which requires dissociation and isolation, CSF cells need to be concentrated to obtain an optimal cell yield. Preserving cell integrity is necessary to prevent the premature degradation of DNA, RNA and proteins and to isolate pure cells with minimal contamination from cell fragments and free nucleic acids. Traditional single-cell isolation methods such as (1) limited dilution where serial dilution techniques are employed and (2) micromanipulation where cells are retrieved via microscope guided capillary pipettes are not useful for isolation of single cells in CSF due to limited cellular yield [27].

A common method of single-cell isolation within CSF is antibody-mediated capture (positive selection) using Flow-Activated Cell Sorting (FACS). In this method, cells are tagged with fluorescent monoclonal antibodies via targeting extracellular membrane proteins for cell sorting [28]. Another single-cell isolation technique, negative selection, involves enriching the desired cell population by removing the known cell population with predetermined fluorescent parameters using electrostatic deflection systems [29]. Both methods require larger CSF volumes and monoclonal antibodies to target proteins of interest. Microfluidic technologies are low cost and allow high-throughput single-cell retrieval from low CSF volumes while minimizing contamination [30]. Commercial platforms such as Fluidigm C1 have been developed for the automation of single-cell lysis and RNA extraction, as well as cDNA synthesis for up to 800 cells on a single chip. However, this requires at least 1000 cells that are relatively homogeneous for capture and downstream processing. Microdroplet-based microfluidics allow for the micro-dispersion of aqueous droplets in a continuous oil phase, enabling high-throughput analysis. Limitations of high-throughput microfluidic technologies are reduced precision, low cell capture efficiency and vitality of cells post-isolation [28].

The CellSearch single-cell capture system, approved by the FDA in 2004 for detecting CTCs in peripheral blood, uses antibodies conjugated to magnets to enumerate CTCs from patient blood samples [31]. Platforms such as CellSearch (Silicon Biosystems) and ClearCell FX (Biolidics) have demonstrated the clinical significance of CTCs, guiding prognostication and holding promise for the advancement of personalized cancer therapeutics. Several novel single-cell isolation strategies including EpCAM-based flow cytometry have shown promise in detecting CTCs [32,33,34]. However, there are no studies combining these platforms with single-cell sequencing to delineate the transcriptomic profile of CTCs. As the field continues to evolve, newer platforms to allow the isolation of single cells of interest from CSF will continue to be developed [28]. A gold standard method of single-cell isolation with high recovery and high specificity can minimize bias and allows a more comprehensive study of the entire cellular landscape. As a result, much attention has been focused on developing improved single-cell isolation techniques.

### 3.3. Library Preparation, Sequencing and Computational Analysis

Library preparation protocols depend on downstream sequencing platforms. Usually, the extracted nucleic acid from each cell is labeled with barcodes then pooled together for sequencing. Several next-generation sequencing methodologies have been developed for single-cell sequencing (Table 1). The advantages and disadvantages of each sequencing technique are beyond the scope of this article. However, the type of library preparation kit, presence of multiple operational steps, prolonged analysis times, low throughout analysis systems, and inexperience of personnel can add variability to results. Simple, high-throughput, inexpensive and reproducible methods are needed to standardize the sequencing process [24,35].

Downstream data processing primarily involves the conversion of raw reads into standardized FASTQ files (text-based files used to store nucleotide sequences), demultiplexing, quality control, filtering, alignment and finally data visualization and interpretation. Several scRNA-seq analysis pipelines have been developed to assess gene expression and interpret single-cell transcriptomics [35]. As discussed, the downstream data largely depends on upstream processing, and this variability should be accounted for when interpreting data. Data analysis should be handled by trained bioinformatics personnel. Finally, it is imperative to have sufficient power to draw reliable and reproducible conclusions.

## 4. Single-Cell RNA Sequencing of the CSF in Neuro-Oncology

CTCs are cancer cells that leave the primary tumor, enter biofluids such as blood or CSF, and play a vital role in tumor dissemination and distant metastasis. Several groups have reported the presence of CTCs in the peripheral blood of patients with GBM [36,37,38,39]. However, reliable detection of glioma CTCs in CSF has remained elusive [40]. In contrast, there is significant evidence of the presence of CSF CTCs in patients with lung cancer and melanoma in the setting of leptomeningeal metastasis [11,41]. Emerging studies have explored the utility of scRNA-seq to study CSF CTCs to understand tumorigenesis, metastasis, and the immune response for both diagnostic and therapeutic purposes (Table 1).

### 4.1. CTC scRNA-Seq to Understand Tumor Heterogeneity, Disease Progression and Metastasis

Ruan and colleagues studied CSF CTCs from patients with leptomeningeal metastasis of adenocarcinoma of the lung [11]. Significant cellular heterogeneity was noted within CTCs isolated from the same patient. scRNA-seq of CSF CTCs in two patients at two different time points showed the possibility of clonal evolution with disease progression. With regard to phenotypic indicators of tumor invasion and metastasis, scRNA-seq of CSF CTCs demonstrated enrichment of genes associated with lung cancer (SFTPB, NAPSA, SLC34A2, SFTA2 and EMP2), disruption of the blood-CSF barrier (MMP7 and C3), cell cycle (CCND1) and epithelial markers (CDH1, EPCAM. KRT7, KRT18, MUC1) [11].

### 4.2. CTC scRNA-Seq to Diagnose the Origin of Carcinoma of Unknown Primary

Ruan and colleagues also used scRNA-seq analysis to explore the possibility of studying CSF to determine the origin of the primary tumor in carcinoma of unknown primary (CUP) based on the gene expression profile of CTCs. Although results were inconclusive, this highlights a future diagnostic avenue using transcriptome analysis of CSF CTCs in patients with leptomeningeal dissemination of CUP to guide diagnosis and therapy [11].

### 4.3. scRNA-Seq to Study the Immune Microenvironment and Metabolic Adaptations of Tumor Cells

Studying CSF immune cells and correlating them with immune cells in the primary tumor site, metastatic site or peripheral blood can provide unique insights into the immune interactions and response in each respective compartment. There is mounting evidence of the ability of cancers to modulate tumor microenvironments into an immune suppressing and tumor supporting system, and scRNA-seq can provide minimally invasive insights into this immune microenvironment. Smalley et al. determined that the CSF immune microenvironment in patients with leptomeningeal metastasis secondary to melanoma was characterized by an immune-suppressed T-cell landscape distinct from that of brain and skin metastases [41]. This study also found that a patient with long-term survival showed a distinct immune repertoire when compared to poor responders. The presence of a rare population of dendritic cells (DC3) was also discovered, which was associated with increased overall survival in patients with melanoma and positively regulated the immune environment through modulation of activated T cells and MHC expression [41].

Chi and colleagues used scRNA-seq to understand how CSF CTCs adapt to microenvironmental challenges including inflammation and sparse micronutrients: breast cancer and non-small cell lung cancer cells in CSF express and use iron-binding protein lipocalin-2 (LCN2) and its receptor SCL22A17 to outcompete macrophages for iron. This may permit tumor cells to evade the immune system by reducing the efficacy of macrophages [42].

Ruan and colleagues studied over a thousand diffuse large B cells in the CSF (CSF-DLBCs) in 6 patients with CNS lymphoma [43]. When compared to normal B cells, gene expression in CSF-DLBCs was enriched in cell proliferation and energy metabolism pathways, which are critical for tumor growth and energy demand. Interestingly, there was downregulation of the antigen processing and presentation pathway and the B cell receptor (BCR) signaling pathway in CSF-DLBCs, suggesting decreased immune cell functionality. There were no transcriptional changes in the B cells in blood and CSF; however, CSF-DLBCs showed differential expression of CCDC167, PTTG1, EML6, EZH2, HAUS1, LAS1L, METTL26, NT5DC2, NCAPH2, NUSAP1, PHF19, PKMYT1, RRM2, SH3TC1, SMC4, and TIMM50, which are genes related to tumor progression, cell cycle state, and cancer-testis antigen expression. This study demonstrates that most CSF-DLBCs demonstrated properties of active cell proliferation and energy metabolism, and inherent heterogeneity which was represented by cell cycle state, CTA expression, and classification by germinal center B-cell signature.

### 4.4. Predicting Responses to Targeted Therapies

scRNA-seq of both CTCs and immune cells in the CSF has provided insights into the immune response to cancer treatment. This may aid in understanding differential responses, diagnosing treatment failure, and providing molecular insights into the adaptations that could be contributing to such failure. A recent study by Mochizuki and colleagues used scRNA-seq of the CSF to evaluate patient response to chimeric antigen receptor (CAR) T-cell therapy for the treatment of H3K27M-mutant diffuse intrinsic pontine glioma (DIPG) and spinal cord diffuse midline glioma (DMG). Patients who demonstrated clinical/radiographic benefit from CAR-T therapy exhibited fewer S100A8 + S100A9+ myeloid suppressor-cells and CD25 + FOXP3+ regulatory T-cells in the CSF pre-infusion when compared to the patient in which a therapeutic response was not achieved. In a subject with DIPG who demonstrated improvement, polyclonal CAR T-cells detectable in CSF at Day +14 demonstrated enrichment of CD8A, GZMA, GNLY and PDCD1 compared to the pre-infusion CAR T-cells by trajectory analysis, suggesting differentiation toward a cytotoxic phenotype. The same subject exhibited increased numbers of S100A8 + S100A9+ myeloid cells and CX3CR1 + P2RY12+ microglia over time. These results provide evidence that the presence of immunosuppressive myeloid populations, detectable in CSF, may correlate with clinical response in CAR T cell therapy for DIPG/DMG [44].

Prakadan and colleagues studied malignant cells and immune cells in patients with leptomeningeal metastasis of solid tumors who underwent treatment with immune checkpoint inhibitors (ICI). An immediate increase in the number of CD8+ T Cells in the CSF following intravenous ICI administration was noted, in addition to increased IFN-γ signaling and cytotoxicity post-treatment, suggesting immunomodulation by ICIs. A decline in immune response to ICIs over time was also noted, emphasizing the value of studying tumor behavior in response to therapy at multiple time points [45]. Similarly, Rubio-Perez and colleagues looked at the T cell landscape in patients with leptomeningeal metastasis of adenocarcinoma of the lung undergoing ICI therapy and identified tumor cells in both the primary tumor and the CSF that exhibited similar genomic profiles. It was also postulated that the primary tumor and leptomeningeal compartments communicate to launch an immune response during ICI therapy, further demonstrating the value of CTC and single cell analysis in understanding the response to therapy [46].

### 4.5. Combined Analysis of CTCs and Other Liquid Biopsy Components

CSF is composed of multiple analytes including circulating free DNA (cfDNA), circulating free RNA (cfRNA), and extracellular vesicles (EVs) in addition to CTCs. cfDNA is released by dying tumor cells, while EVs are actively secreted by tumor cells as molecular messengers [23,47]. Using multiple liquid biopsy analytes for comparison in scRNA-seq may provide additional insights into disease progression and response to therapy. Li and colleagues studied both CTCs and cfRNA to identify lung-specific expression profiles in patients with leptomeningeal dissemination from non-small cell lung cancer (NSCLC) [48]. Specifically, the gene encoding CEACAM6 was identified in both CTCs and cfRNA, which has several implications in NSCLC migration. Thus, developing standardized protocols which enable isolating and studying multiple liquid biopsy analytes from blood and CSF can provide robust diagnostic and monitoring insights.

## 5. Single-Cell RNA Sequencing in CNS Disease of Non-Oncological Etiology

Studying the cellular landscape of CSF has been an avenue of interest in several non-oncologic diseases where routine biopsy is not obtained or feasible. Emerging studies have used scRNA-seq to gain insights into disease progression and neuroinflammation (Table 1).

### 5.1. Alzheimer’s Disease

Alzheimer’s disease (AD) is a neurodegenerative disorder characterized by progressive cognitive impairment without a definitive cure. There has been significant interest in understanding the immune response and its contribution to AD progression. scRNA-seq of CSF in patients with AD revealed that T-cell receptor (TCR) signaling was upregulated in circulating CSF CD8+ T- effector memory CD45RA+ (TEMRA) cells, which was negatively associated with patient cognition [49]. Gate and colleagues first used mass cytometry to analyze peripheral blood mononuclear cells of patients with Alzheimer’s to show an increased number of CD8+ TEMRA cells, which were negatively associated with cognition. Additionally, scRNA sequencing of these cells revealed an enhanced T cell receptor (TCR) signaling. Interestingly, similar clonally expanded CD8+ TEMRA cells were also found in CSF, which reveals an adaptive immune response in the blood as well as CSF in AD providing evidence of clonal, antigen-experienced T cells patrolling the intrathecal space of brains affected by neurodegeneration. These TCRs notably had specificity for two separate Epstein-Barr virus (EBV) antigens; however, there is no evidence of a causal relationship [49]. Thus, while several pathologically significant protein-based CSF biomarkers such as amyloid-b (Ab42), total tau (T-tau), and phosphorylated tau (P-tau) have been traditionally used for AD diagnostics, cellular CSF biomarkers may provide novel insights into the immune responses [50].

### 5.2. Neurological Sequelae of COVID-19

scRNA-seq has also been employed to investigate the mechanism by which some patients develop neurological symptoms such as anosmia, dysgeusia, headache, impaired consciousness, and seizures as a result of COVID-19 infection. Song and colleagues studied the CNS immune response in individuals with neurological sequelae of COVID-19 using scRNA-seq and cytokine analyses of CSF and blood, finding evidence of CNS-specific T cell activation and B cell responses distinct from peripheral responses during infection. In addition to uncovering the transcriptional profiles of CSF immune cells in patients with neurological symptoms of COVID-19 infection, it was found that typical interactions between CD4+ T cells and monocytes were diminished, suggesting a dysregulated innate-to-adaptive immune interface [51].

### 5.3. Multiple Sclerosis

Multiple sclerosis (MS) is an autoimmune demyelinating disease of the CNS with ongoing research using scRNA-seq to elucidate the etiology and potential treatments of aberrant immune activation. scRNA-seq of immune cells in the CSF has provided evidence that in MS, CSF B cells are driven to inflammatory and clonally expanded memory and plasmablast/plasma cell phenotypes [52]. scRNA-seq has also permitted the identification of distinct myeloid cell types present within the CSF of subjects with neuroinflammation resulting from MS, Anti-MOG disorder and HIV [53]. Beltrán et al. used scRNA-seq of CSF cells to provide evidence for the early activation of the adaptive immune system in monozygotic twins with prodromal MS, with notable activity of clonally expanded TRM-like CD8+ cells, plasmablasts, and CD4+ T cells [54].

### 5.4. HIV-Associated Neuronal Injury

Researchers have been studying the role of CNS immune activation in patients with neurocognitive impairment in the setting of HIV, even during sustained long term suppressive antiretroviral therapy. Farhadien and colleagues used scRNA-seq of CSF and blood from adults with and without HIV to identify a rare subset of myeloid cells with a genetic signature that overlaps significantly with neurodegenerative disease-associated microglia. CNS myeloid cells and microglia in particular have been proposed to play several important roles in HIV infection, including acting as a potential site of viral replication [55].

### 5.5. Meningitis

Researchers have begun using scRNA-seq to study the CSF cellular landscape in patients with bacterial meningitis that is unresponsive to antibiotic treatment [56]. Chen et al. used scRNA-seq to identify the infectious agent causing meningitis in a 31-year-old male with atypical presentation [56]. Initial tests were inconclusive as *Cryptococcus* cell culture was negative, while the Cr-Ag agglutination test was weakly positive (1:1). Thus, scRNA-seq of the CSF was employed to identify the etiological agent: *C. gattii sensu stricto.* This case highlights the applicability of scRNA-seq in establishing the etiology of rarer causes of meningitis when initial testing is inconclusive.

## 6. Limitations

Circulating cells in CSF are relatively difficult to isolate due to low concentration in the CSF, necessitating larger volumes of CSF to obtain adequate cellular yield. However, ex vivo culture of circulating cells can generate patient-specific disease models to allow individualized treatment planning. Another limitation of scRNA-seq of the CSF that has been touched upon throughout the manuscript is inherent to the novelty of this technology: because the field is still relatively new, definitive standards for quality control, data processing, and interpretation have yet to be established and implemented. Such standardization will be necessary to ensure results are reproducible and translatable from benchtop cell cultures to bedside diagnostics. Available studies have limitations including small sample sizes and variability in processing, sequencing, and downstream analysis (Table 1). Thus, larger studies with well-defined cohorts and standardized protocols are needed to gain more reliable insights into CSF cellular biology and its role in disease.

## 7. Conclusions

Rapid technological development and improvement in high-throughput single-cell sequencing, together with the methodological advances in single-cell isolation and downstream processing of CSF in patients with neurological diseases, have advanced the field of liquid biopsy. In this review, we presented studies that leverage scRNA-seq of CTCs and immune cells in the CSF which identify unique cell populations and characterize the immune microenvironment to diagnose and treat both neoplastic and non-neoplastic diseases of the CNS. While standardized protocols must be developed and implemented for scRNA-seq technology to be consistently translatable to clinical practice, this field holds tremendous promise for new diagnostic methods and targeted individualized treatments.

## Figures and Tables

**Table 1:** Studies using scRNA-seq of CSF cells for the diagnosis and investigation of neurological disease.

Disease	Study	Experimental Group	No. Patients	Control Group	No. Patients	Cell Isolation Method	Sequencing Method	Conclusion
LMM	Ruan et al. (2020)	Patients with LMM secondary to LUAD or CUP	5	Healthy controls	3	FACS	Smart-seq2/Illumina HiSeqX	Identified candidate genes for RNA-based detection of CSF-CTCs in patients with LMM of LUAD and CUP.
LMM	Chi et al. (2020)	Patients with LMM secondary to breast cancer (N = 3) or NSCLC (N = 2)	5	Patients with cancer having no LMM	18	Flow cytometry	10X Genomics/Illumina NovaSeq 6000	Cancer cells, but not macrophages, within the CSF of patients with LMM express the iron-binding protein LCN2 and its receptor SCL22A17, with cancer cells appearing to survive in the CSF by outcompeting macrophages for iron.
LMM	Li et al. (2021)	Patients with LMM secondary to NSCLC	4	Nontumorgenic cells (e.g., immune cells) in the CSF	1	Microfluidic chip	Nextera XT DNA Library Preparation Kit (Illumina)/NextSeq mid-output Kit (Illumina)	Cell migration in NSCLC cell lines was directly proportional to CEACAM6 expression, suggesting a role in disease progression.
LMM	Prakadan et al. (2021)	Patients enrolled in clinical trials investigating the use of ICI in LMD	19	Nontumorgenic cells (e.g., immune cells) in the CSF	19	Pico-wells	Seq-Well/Illumina 75 Cycle NextSeq500 Kit or Illumina 100 Cycle NovaSeq6000S Kit	CD8+ T cells in the CSF are more abundant and proliferative in samples treated with ICI and exhibited higher levels of genes associated with effector function and IFN-γ signaling relative to untreated samples.
Brain metastasis and LMM	Rubio-Perez et al. (2021)	Patients with brain metastasis or LMM of LUAD, LUSC, SCLC, SKCM, BRCA, ESCA, HNSC and URO	6	Tumor-infiltrating cells as opposed to CSF cells	6	Flow cytometry	10X Genomics/Illumina NovaSeq 6000	Brain metastasis immune cell infiltrates are recapitulated in the CSF compartment. There was a significantly higher CD8+/CD4+ T cell ratio in the tumor compared to the CSF.
Brain metastasis and LMM	Smalley et al. (2021)	Patients with brain metastasis or LMM of melanoma	24	Patients with skin metastasis only	2	NR	10X Genomics/Illumina NextSeq 500	The LMM microenvironment was characterized by an immune-suppressed T-cell landscape distinct from that of brain and skin metastases. A rare population of dendritic cells (DC3) was associated with increased overall survival and positively regulated the immune environment through modulation of activated T cells and MHC expression.
CNSL-DLBCL	Ruan et al. (2021)	Patients with LMM secondary to CNSL-DLBCL	6	Healthy controls	3	FACS	Smart-seq2/Illumina HiSeqX	Identified inherent heterogeneity of CSF-DLBCs in cell cycle state, cancer-testis antigen expression, and classification based on single-cell germinal center B-cell signature. Identified 16 upregulated genes in CSF-DLBCs compared to normal B cells, which showed possible ‘homing effect’ of the CNS-DLBCL for the leptomeninges.
Alzheimer’s Disease	Gate et al. (2020)	Patients with AD or prodromal MCI	9 AD, 9 MCI	Age-matched healthy controls	9	Flow cytometry	10X Genomics/NextSeq550 Sequencer (Illumina)	TCR signaling was enhanced in CD8+ TEMRA cells circulating in the CSF of patients with AD and was negatively associated with patient cognition.
Neurological sequelae of COVID-19	Song et al. (2021)	Patients with neurological sequelae of COVID-19 infection	6	Uninfected controls	3	Flow cytometry	10X Genomics/Illumina Novaseq	Immune cell scRNA-Seq showed divergent T cell activation in the CNS during COVID-19 infection.
Multiple Sclerosis	Beltrán et al. (2019)	Twins with multiple sclerosis or subclinical neuroinflammation	16	Non-MS twins and control patients with idiopathic intracranial hypertension	6	FACS	Smart-Seq2/Illumina NGS HiSeq	Provided evidence for early concomitant activation of 3 components of the adaptive immune system in MS, with a notable contribution of clonally expanded TRM-like CD8+cells.
Multiple Sclerosis	Ramesh et al. (2020)	Patients with RRMS (n = 12) or other neurologic disease (n = 1)	13	Healthy controls	3	Flow cytometry	10X Genomics/Illumina HiSeq4000	Provided evidence that in MS, CSF B cells are driven to an inflammatory and clonally expanded memory and plasmablast/plasma cell phenotype.
Multiple Sclerosis, Anti-MOG disorder, HIV	Esaulova et al. (2020)	Patients with inflammatory demyelinating disease (either RRMS and or anti-MOG disorder), available sequencing data on 2 patients with HIV	13	A subject with IIH, a subject with ALS, and a healthy control	3	Flow cytometry	10X Genomics/Illumina HiSeq4000 or Novaseq Sequencer	Identified distinct myeloid cell types present within the CSF of subjects with neuroinflammation.
Multiple Sclerosis	Hrastelj et al. (2021)	Patients with newly diagnosed, treatment-naïve MS	21	Patients with non-inflammatory disorders (e.g., IIH)	20	FACS	Tecan Ovation SoLo RNA-seq System/Illumina HiSeq4000	CSF CD4+ T cells displayed a distinct gene expression profile when compared to blood CD4+ T cells, which was similar in non-inflammatory controls and MS and was predominated by migration molecules.
HIV	Farhadian et al. (2018)	HIV+ participants	3	Non-HIV+ controls	2	SeqWell array	SeqWell/Illumina HiSeq4000 platform	Identified a rare (<5% of cells) subset of myeloid cells found only in the CSF that present a gene expression signature that overlaps significantly with neurodegenerative disease–associated microglia and may perpetuate neuronal injury during HIV infection.
Meningitis	Chen et al. (2020)	Patient with cryptococcal meningitis (case report)	1	N/A	N/A	Laser microdissection	REPLI-g Single Cell Kit/NEBNext^®^ UltraDNA Library Prep Kit	scRNA sequencing was used for the diagnosis of CNS-related mycosis caused by pathogenic fungi that could not be cultured.

Abbreviations: AD: Alzheimer’s disease; ALS: amyotrophic lateral sclerosis; BRCA: breast carcinoma; CNSL: central nervous system lymphoma; CSF: cerebrospinal fluid; CTC: circulating tumor cell; CUP: carcinoma of unknown primary; DLBCL: diffuse large B cell lymphoma; ESCA: esophageal carcinoma; FACS: Fluorescence-Activated Cell Sorting; HIV: human immunodeficiency virus; HNSC: head and neck squamous carcinoma; ICI: immune checkpoint inhibitor; IIH: idiopathic intracranial hypertension; LCN2: lipocalin-2; LMD: leptomeningeal disease; LMM: leptomeningeal metastasis; LUAD: lung adenocarcinoma; LUSC: lung squamous carcinoma; MCI: mild cognitive impairment; MOG: myelin oligodendrocyte glycoprotein; MS: multiple sclerosis; N/A: not applicable; NR: not reported; NSCLC: non-small cell lung cancer; RRMS: relapse-remitting multiple sclerosis; SCLC: small cell lung cancer; scRNA-Seq: single-cell RNA sequencing; SKCM: skin cutaneous melanoma; TCR: T cell receptor; TEMRA: terminally differentiated effector memory T cells; TRM: resident memory T cells; URO: urothelial carcinoma.

## Data Availability

Not applicable.
